# Thermal Degradation of Linalool-Chemotype *Cinnamomum osmophloeum* Leaf Essential Oil and Its Stabilization by Microencapsulation with β-Cyclodextrin

**DOI:** 10.3390/molecules26020409

**Published:** 2021-01-14

**Authors:** Hui-Ting Chang, Chun-Ya Lin, Li-Sheng Hsu, Shang-Tzen Chang

**Affiliations:** 1 School of Forestry and Resource Conservation, National Taiwan University, Taipei 10617, Taiwan; r05625034@ntu.edu.tw (L.-S.H.); peter@ntu.edu.tw (S.-T.C.); 2 Department of Wood Based Materials and Design, National Chiayi University, Chiayi 600355, Taiwan; keocylin@mail.ncyu.edu.tw

**Keywords:** *Cinnamomum osmophloeum*, linalool, β-cyclodextrin, microencapsulation

## Abstract

The thermal degradation of linalool-chemotype *Cinnamomum osmophloeum* leaf essential oil and the stability effect of microencapsulation of leaf essential oil with β-cyclodextrin were studied. After thermal degradation of linalool-chemotype leaf essential oil, degraded compounds including β-myrcene, *cis*-ocimene and *trans*-ocimene, were formed through the dehydroxylation of linalool; and ene cyclization also occurs to linalool and its dehydroxylated products to form the compounds such as limonene, terpinolene and α-terpinene. The optimal microencapsulation conditions of leaf essential oil microcapsules were at a leaf essential oil to the β-cyclodextrin ratio of 15:85 and with a solvent ratio (ethanol to water) of 1:5. The maximum yield of leaf essential oil microencapsulated with β-cyclodextrin was 96.5%. According to results from the accelerated dry-heat aging test, β-cyclodextrin was fairly stable at 105 °C, and microencapsulation with β-cyclodextrin can efficiently slow down the emission of linalool-chemotype *C. osmophloeum* leaf essential oil.

## 1. Introduction

Natural products from cinnamon plants (*Cinnamomum* spp., Lauraceae) exhibit various bioactivities, such as antimicrobial, insecticidal, anti-inflammatory, antidiabetic activities and, etc. [1,2,3,4,5]. *Cinnamomum osmophloeum* Kanehira is commonly used as folk medicines and food flavors. It is scientifically reported that extracts and essential oils of *C. osmophloeum* exhibit the antioxidant, antibacterial, anxiolytic, xanthine oxidase inhibitory effects, and so forth [6,7,8,9].

Parts of natural plant products, especially essential oils, are highly volatile in the ambient environment and, therefore, may result in thermal oxidation/degradation. Microencapsulation or nanoencapsulation of the essential oils and extracts could provide protection and enhance the stabilization [10,11,12]. Encapsulation materials used include gelatin, cyclodextrins, gum arabic, caseinates, alginates, cellulose derivatives, chitosans, etc. [13,14,15,16]. Properties of natural plant products after microencapsulation would be influenced by the kinds of core-shell structures and coating materials [17,18,19,20].

Cyclodextrins are amphiphilic hollow cyclic oligosaccharides and form the inclusion complexes with versatile molecules [17,21,22]. β-Cyclodextrin, composed of seven α-d-glucopyranose units, is the most common cyclodextrin product and of good durability; it is a multifunctional encapsulation material to keep the bioactive ingredients from volatilization, oxidization, and etc. [23,24,25]. Many researchers have reported the microencapsulation of the bioactive constituents, essentials oil, or supercritical fluid extracts with β-cyclodextrin [9,10,26,27]. Ramos et al. proved that the inclusion of isopulegol, an alcoholic monoterpene, in β-cyclodextrin is an effective method to improve its antiedematogenic and anti-inflammatory activities [28]. Cyclodextrins are appropriate carriers for delivering or releasing natural products in the pharmaceutical, food and cosmetic industries [16,29,30,31,32,33].

The aims of this study were to investigate the thermal degradation of linalool-chemotype *C. osmophloeum* leaf essential oil, find the optimal reaction conditions of microencapsulation of leaf essential oil with β-cyclodextrin, and evaluate the stabilization of leaf essential oil microcapsules. Through our research, it is expected to reveal the changes in the chemical structure of linalool during thermal decomposition and properly preserve the linalool-chemotype *C. osmophloeum* leaf essential oil by microencapsulating with β-cyclodextrin.

## 2. Materials and Methods

### 2.1. Hydrodistillation

Fresh *C. osmophloeum* leaves were collected from Lienhuachih Research Center of Taiwan Forestry Research Institute in Nantou County, Taiwan. Leaves were hydrodistilled in a Clevenger apparatus for 6 h to obtain the leaf essential oil. The yield of leaf essential oil was 3.46 ± 0.06% (*w/w*). Leaf essential oil was stored in the dark glass bottle and kept in the refrigerator at 4 °C.

### 2.2. GC–MS Analysis

The constituents of the leaf essential oil were analyzed by Thermo Trace GC Ultra gas chromatograph equipped with a Polaris Q MSD mass spectrometer (Thermo Fisher Scientific, Austin, TX, USA). Each 1 μL analyte was injected into the DB-5MS capillary column (Crossbond 5% phenyl methyl polysiloxane, 30 m length × 0.25 mm i.d. × 0.25 µm film thickness). The temperature program was: 60 °C initial temperature for 1 min; 4 °C/min up to 220 °C and hold for 2 min; and 20 °C/min up to 250 °C and hold for 3 min. The flow rate of carrier gas helium was 1 mL/min, and the split ratio was 1:10. Constituents were identified by comparing the mass spectra (*m*/*z* 50–600 amu) with NIST and Wiley library data, Kovats index (KI) [34] and authentic standards. Quantification of constituents was analyzed by integrating the peak area of the chromatogram using GC equipped with the flame ionization detector (FID).

### 2.3. Microencapsulation

Leaf essential oil microencapsulated with β-cyclodextrin was using the co-precipitation method with slight revisions [35,36,37]. β-Cyclodextrin (5 g) was first dissolved in 300 mL different ratio of ethanol/water solution at 50 °C for 5 min; the solution was cooled down to 25 °C. Linalool/leaf essential oil (0.88 g) was dissolved in 10 mL ethanol; and then added to the β-cyclodextrin solution, stirred at 600 rpm for 1 h. The solution was kept in the refrigerator at 4 °C overnight; the co-precipitated microcapsules were filtered and then washed with 50 mL distilled water. Microcapsules were dried at 50 °C in the oven for 24 h. The yield of leaf oil microcapsules was calculated by the following Formula (1).

Yield (%) = microcapsules (g)/[β-cyclodextrin (g) + leaf essential oil (g)] × 100(1)

### 2.4. Accelerated Dry-Heat Aging Test

An accelerated dry-heat aging test (ISO 5630–1; CNS 12,887–1) was used to evaluate the thermostability of leaf oil microcapsules. Microcapsules were heated at 105 °C in a ventilated oven. Weights of leaf oil microcapsules were measured during the accelerated dry-heat aging test (1, 2, 4, and 8 days). After the aging test, weight losses of leaf oil microcapsules were calculated.

### 2.5. Statistical Analysis

Results data were statistically analyzed using Scheffé’s test of the SAS system (version 9.2, Cary, NC, USA) with a 95% confidence interval. Scheffé’s test is a post hoc multiple comparison method with stringent error control.

## 3. Results and Discussion

### 3.1. Changes in Composition of C. osmophloeum Leaf Oil after Thermal Degradation

**Figure 1:**
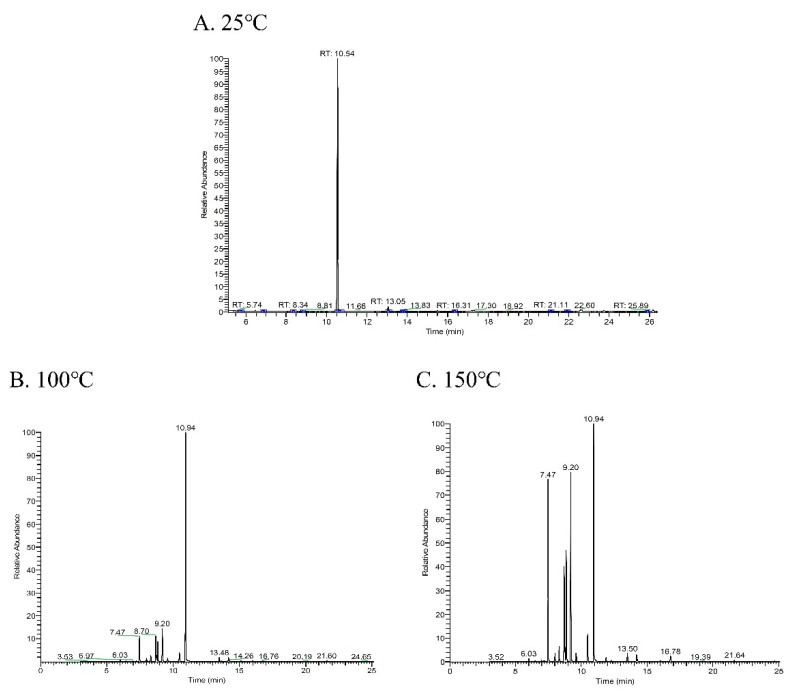
Gas chromatogram of linalool-chemotype *C. osmophloeum* leaf oil after thermal degradation. (**A**) 25 °C; (**B**) 100 °C; (**C**) 150 °C.

Constituents of *C. osmophloeum* leaf essential oil were analyzed by GC–MS; a gas chromatogram of leaf essential oil is shown in Figure 1A. The main constituent of leaf essential oil was linalool (93.30%), the other minor constituents were 2-methyl benzofuran (1.99%), α-pinene (0.66%), cinnamyl acetate (0.63%), limonene (0.61%), β-caryophyllene (0.59%), methyl chavicol (0.57%), and *trans*-cinnamaldehyde (0.52%), as listed in Table 1. Due to the high content of linalool, *C. osmophloeum* leaf essential oil was classified into the linalool-chemotype.

As presented in Figure 1B, several significant peaks occurred in the gas chromatogram of leaf essential oil after the heat treatment at 100 °C for 30 min. The content of the main constitute, linalool, was reduced from 93.30% to 64.01% (Table 1). New constituents were observed for β-myrcene (5.56%), α-phellandrene (0.91%), α-terpinene (1.49%), *cis*-ocimene (4.70%), γ-terpinene, and terpinolene (2.53%) in the thermally degraded leaf essential oil. Major variations were found for the increasing contents of limonene and *trans*-ocimene, which were 0.61% and 0.32% in the raw leaf essential oil and obviously increased to 7.77% and 7.94% in the thermally degraded specimen.

**Figure 2:**
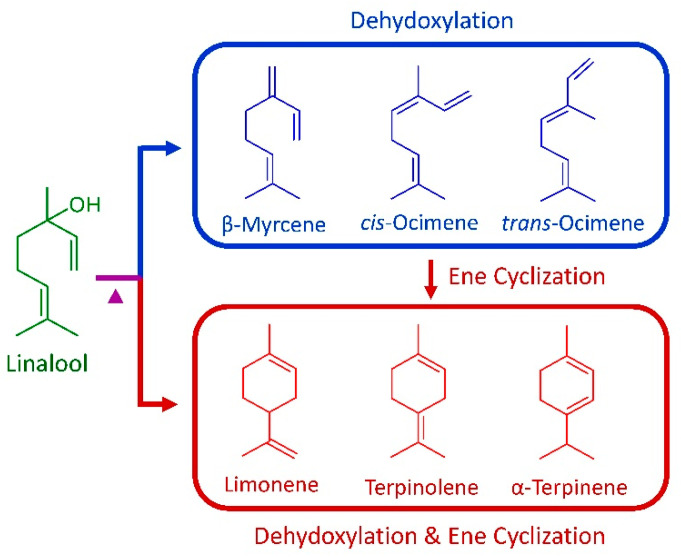
Schematic illustration of the thermal degradation of linalool.

Similar degradation was observed from the gas chromatogram of leaf essential oil after the heat treatment at 150 °C for 30 min (Figure 1C). Linalool had a remarkable decrease in content from 93.30% in the original leaf essential oil to 27.54% in the 150 °C-degraded specimens. The peaks of the new compounds generated under more severe heat treatment became more obvious; *trans*-ocimene was present in the degraded leaf essential oil of 20.08%, β-myrcene 17.89%, *cis*-ocimene 11.72%, limonene 11.40%, terpinolene 3.37%, and α-terpinene 1.69%, in comparison with the original leaf essential oil, where these contents were much smaller. The increased amount (65.22%) of these compounds was close to the decrease in linalool (65.76%). Figure 2 illustrates the degradation mechanism of linalool and chemical structures of degradation products. After heat treatments at 100 °C and 150 °C, compounds β-myrcene, *cis*-ocimene and *trans*-ocimene were formed through the dehydroxylation of linalool. Moreover, ene cyclization occurred to linalool and its dehydroxylated products, further formed the compounds limonene, terpinolene and α-terpinene.

Leiner et al. (2013) investigated the pyrolysis behavior of linalool. Linalool was pyrolyzed in a temperature range of 350–600 °C under nitrogen and underwent ene-type cyclization reactions leading to plinols, four cyclopentanol compounds [38]. The result varied from this study may be due to the different heating temperatures and the environment (under N2 or under air).

### 3.2. Optimization of Microencapsulation of Leaf Essential Oil with β-Cyclodextrin

The preparation method of microencapsulation can influence the property of β-cyclodextrin microcapsules. Kfoury et al. (2016) studied the aroma release effect from the solid inclusion complexes of β-cyclodextrin with trans-anethole by two preparation methods, freeze-drying (FD) and co-precipitation coupled to FD (Cop-FD). Cop-FD microcapsules retained more efficiently trans-anethole than that of FD microcapsules; it revealed co-precipitation method provide superior inclusion characteristics [12].

The specimen to β-cyclodextrin ratio and the solvent ratio is the important factors that influence the yield of microcapsules. Yields of linalool and leaf essential oil microencapsulated with β-cyclodextrin by different reaction conditions are presented in Table 2. β-Cyclodextrin completely dissolved in the solution (ethanol/water, 1:2 *v/v*) by heating at 50 °C for 5 min, then the solution was cooled down to 25 °C without adding the core material, and no powders/crystals formed or precipitated even at 4 °C. The highest yield of microcapsule was 94.2% at the linalool to the β-cyclodextrin ratio of 15:85 (*w/w*), which was quite close to the molar ratio (linalool:β-cyclodextrin) of 1:1.

As for the ethanol/water ratio, the highest yield of microcapsule was 98.1% under the 1:5 ratio of ethanol to water. There was no statistically significant difference in the microcapsule yields between the solvent ratio of 1:3 and 1:5 (*p* < 0.05). Using the optimal reaction conditions, the yield of linalool-chemotype leaf essential oil microencapsulated with β-cyclodextrin was 96.5%.

### 3.3. Stabilization and Release of Leaf Essential Oil Microcapsules

The constituents of common essential oils from aroma plants are small molecular weight and highly volatile. The encapsulation of limonene would influence its properties, such as evaporation, stabilization and controlled release, by different encapsulation methods and selected materials. The retention of limonene in extruded starch (non-encapsulation) was quite low (8.0%) compared with that of limonene microencapsulated with β-cyclodextrin (92.2%) [20].

**Figure 3:**
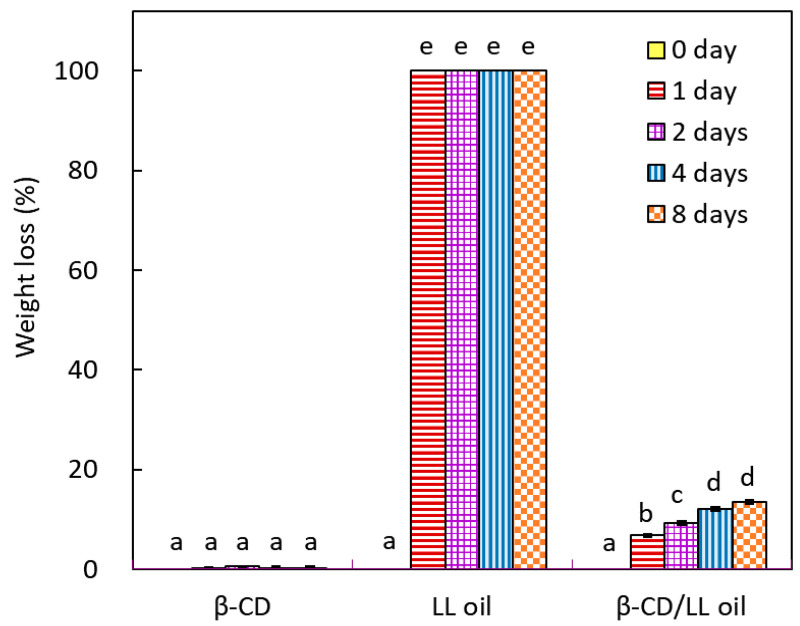
Changes in weight loss of leaf essential oil microcapsules during the dry-heat aging period. β-CD: β-cyclodextrin; LL oil: *C. osmophloeum* leaf oil; β-CD/LL oil: leaf oil microcapsules; different letters (a–e) in the figure refer to the statistically significant difference at the level of *p* < 0.05 according to the Scheffé’s test.

Using the accelerated dry-heat aging test to evaluate the stability and release of leaf essential oil microcapsules. Figure 3 shows the weight losses of β-cyclodextrin (β-CD), linalool-chemotype leaf essential oil (LL oil), and leaf oil microcapsules (β-CD/LL oil) at 105 °C during the accelerated aging period. The weight loss of β-CD was very slight (less than 0.5%) after 8 days of the accelerated aging test; it indicated that β-CD was thermostable at 105 °C. Trotta et al. (2000) investigated the thermal behavior of β-CD; the decomposition temperature of β-cyclodextrin was 250 °C by using the thermogravimetric analysis (TGA) [39]. Weight losses of linalool-chemotype leaf essential oil was 99.1% after 30 min at 105 °C (data not shown in Figure 2); leaf essential oil exhibited highly volatile in the high-temperature environment. Weight losses of leaf essential oil microcapsules were 6.73%, 9.33%, 12.14%, and 13.40% after 1, 2, 4, and 8 days of the aging test, respectively. Results revealed that microencapsulation with β-cyclodextrin slowed down the release/emission of leaf essential oil in the dry-heat aging test and improved the thermal stabilization of linalool-chemotype leaf essential oil.

## 4. Conclusions

The thermal degradation of linalool-chemotype *C. osmophloeum* leaf oil is investigated by GC–MS. After the heat treatment, compounds β-myrcene, cis-ocimene and trans-ocimene form through the dehydroxylation of linalool and compounds limonene, terpinolene and α-terpinene by a further ene cyclization. The significantly high microcapsule yield of 96.5% is obtained from the optimal reaction conditions with the leaf essential oil to the β-cyclodextrin ratio of 15:85 and ethanol to water ratio of 1:5. Based on the accelerated dry-heat aging assay, β-cyclodextrin is stable under the environment at 105 °C, and microencapsulation with β-cyclodextrin effectively slows down the release/emission of leaf essential oil.

## Figures and Tables

**Table 1:** Compositions of linalool-chemotype *Cinnamomum osmophloeum* leaf essential oil after thermal degradation.

Rt	KI	rKI	Constituent	Content (%)		
(min)				Original	100 °C	150 °C
6.03	938	939	α-Pinene	0.66	0.36	0.29
7.21	982	979	β-Pinene	0.28	0.07	0.06
7.47	991	990	β-Myrcene	-	5.56	17.89
8.01	1007	1002	α-Phellandrene	-	0.91	0.98
8.32	1017	1017	α-Terpinene	-	1.49	1.69
8.70	1027	1029	Limonene	0.61	7.77	11.40
8.86	1032	1037	*cis*-Ocimene	-	4.70	11.72
9.20	1049	1050	*trans*-Ocimene	0.32	7.94	20.08
9.60	1055	1059	γ-Terpinene	-	0.86	0.97
10.48	1082	1088	Terpinolene	-	2.53	3.37
10.94	1100	1096	Linalool	93.30	64.01	27.54
13.48	1180	-	2-Methylbenzofuran	1.99	1.23	1.07
14.17	1186	1188	α-Terpineol	0.28	1.50	1.12
14.28	1198	1196	Methyl chavicol	0.57	-	-
16.80	1273	1270	*trans*-Cinnamaldehyde	0.52	0.15	0.74
21.64	1420	1424	β-Caryophyllene	0.59	0.22	0.17
22.42	1445	1446	Cinnamyl acetate	0.63	0.02	-

RT: retention time (min); KI: Kovats index relative to *n*-alkanes (C9 – C24) on a DB-5MS column; rKI: Kovats index on a DB-5MS column in the reference [34].

**Table 2:** Yields of linalool and leaf essential oil microencapsulated with β-cyclodextrin.

Specimen	Specimen: β-CD (*w/w*)	EtOH: H_2_O (*v/v*)	Yield (%)
	0:100	1:2	0.0 ± 0.0 ^d^*
	5:95	1:2	54.3 ± 3.1 ^a^*
Linalool	10:90	1:2	86.9 ± 0.2 ^b^
	15:85	1:2	94.2 ± 0.4 ^c^
	20:80	1:2	91.0 ± 0.5 ^b,c^
	15:85	1:7	93.3 ± 0.6 ^B^
	15:85	1:5	98.1 ± 0.1 ^C^
Linalool	15:85	1:3	97.0 ± 0.4 ^C^
	15:85	1:2	94.2 ± 0.4 ^B^
	15:85	1:1	83.4 ± 1.8 ^A^
Leaf essential oil	15:85	1:5	96.5 ± 0.2

*: Different letters (^a–d^ and ^A–C^) in Table refer to statistically significant difference at the level of *p* < 0.05 according to Scheffé’s test.

## Data Availability

The data are available are available from the corresponding author on reasonable request.

## Sample Availability

Samples are available the corresponding author.
